# Meta-analysis of Soy Consumption and Gastrointestinal Cancer Risk

**DOI:** 10.1038/s41598-017-03692-y

**Published:** 2017-06-22

**Authors:** Demin Lu, Chi Pan, Chenyang Ye, Huijie Duan, Fei Xu, Li Yin, Wei Tian, Suzhan Zhang

**Affiliations:** 1 Cancer Institute (Key Laboratory of Cancer Prevention and Intervention, China National Ministry of Education), Second Affiliated Hospital, School of Medicine, Zhejiang University, Hangzhou, Zhejiang China; 2 Department of Medical Oncology, Second Affiliated Hospital, School of Medicine, Zhejiang University, Hangzhou, Zhejiang China; 3 Reseach Center for Air Pollution and Health, School of Medicine, Zhejiang University, Hangzhou, Zhejiang 310009 China

## Abstract

Soy consumption has received considerable attention for its potential role in reducing cancer incidence and mortality. However, its effects on gastrointestinal (GI) cancer are controversial. Therefore, we performed a meta-analysis to evaluate the association between soy consumption and gastrointestinal cancer risk by searching for prospective studies in PubMed, Web of Science, EMBASE and the reference lists of the included articles. The study-specific odds ratio (OR), relative risk (RR) or hazard ratio (HR) estimates and 95% confidence intervals (CIs) were pooled using either a fixed-effect or random-effect model. Twenty-two independent prospective studies were eligible for our meta-analysis, including 21 cohort studies and one nested case-control study. Soy product consumption was inversely associated with the incidence of overall GI cancer (0.857; 95% CI: 0.766, 0.959) and the gastric cancer subgroup (0.847; 95% CI: 0.722, 0.994) but not the colorectal cancer subgroup. After stratifying the results according to gender, an inverse association was observed between soy product intake and the incidence of GI cancer for females (0.711; 95% CI: 0.506, 0.999) but not for males.

## Introduction

In recent years, soy consumption has received considerable attention for its potential role in reducing the incidence and mortality of cancer^1–5^. Much literature has studied the possible association between soy consumption and gastrointestinal (GI) cancer^4, 6–8^. The lower risk of GI cancer that results from a greater soy intake may be explained through multiple biological effects, including inflammation inhibition, antioxidant activity, anti-proliferative properties and angiogenesis^9–11^.

However, population studies of the association between soy intake and GI cancer risk have yielded inconsistent results. In 2016, Umesawa *et al*. reported that the consumption of large quantities of miso soup was associated with an increased risk of gastric cancer among the Japanese population^12^. In 2015, Wada *et al*. reported that the higher intake of soy foods was significantly associated with a lower risk of stomach cancer^6^. Some recent meta-analyses reported that the consumption of soy was inversely associated with gastric cancer^13, 14^, while in 2016, Tse *et al*. reported that there was no association between soy intake and gastric cancer^15^.

Previous meta-analysis studies on this topic combined both retrospective case-control studies and prospective cohort studies. To overcome the shortcomings of the retrospective studies, such as the likelihood of exposure to recall bias and selection bias, we investigated the association between soy intake and GI cancer only in prospective studies.

## Results

### Literature search

**Figure 1:**
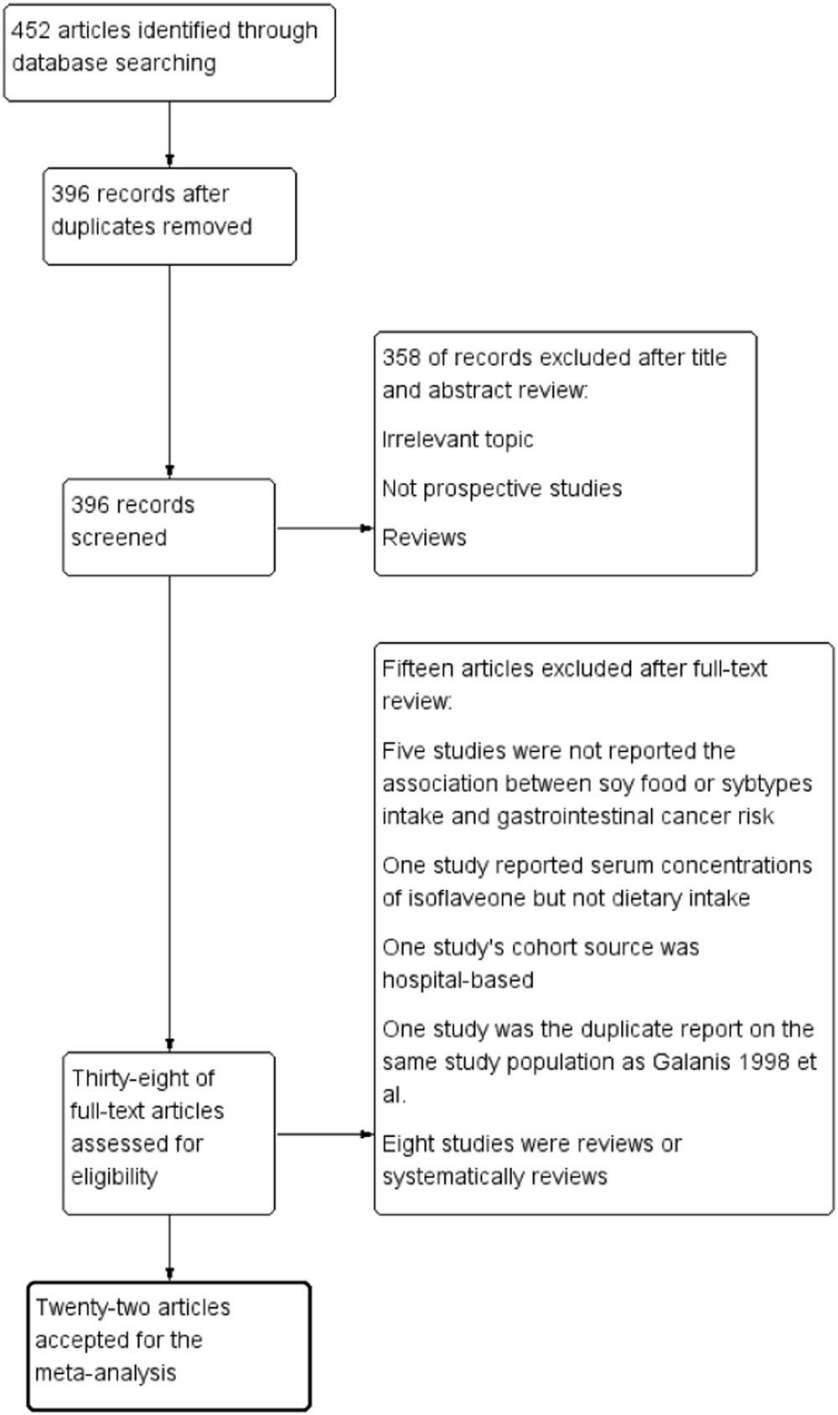
Flow diagram of selection process for inclusion studies in the meta-analysis of soy consumption and GI cancer risk.

The literature search through PubMed, Web of Science and EMBASE identified a total of 452 abstracts. After removing duplicates, 396 abstracts remained. The title and abstract screening excluded 358 articles. Thus, we identified 38 potentially relevant studies. The entire text of all remaining studies was reviewed, and 15 studies were excluded for the following reasons: five studies did not report the association between the intake of soy food or its subtypes and gastrointestinal cancer risk^7, 16–19^, one study reported serum concentrations of isoflavone but not dietary intake^8^, one study’s cohort source was hospital-based^20^, one study was a duplicate report on the same study population that Galanis *et al*. (1998) used^21^, and eight studies were either reviews or systematic reviews^14, 15, 22–27^. Therefore, twenty-two independent prospective studies were eligible for our meta-analysis, including 21 cohort studies^6, 12, 28–46^ and one nested case-control study^47^. The flow diagram of our systematic literature search is shown in Fig. 1.

### Study characteristics

The characteristics of the eligible studies are outlined in Table 1. We included 22 independent studies that contained a total of 12,901 cancer cases from 965,466 participants. Fifteen studies reported the association between soy consumption and gastrointestinal cancer incidence, while seven studies reported the association between soy consumption and gastrointestinal cancer mortality. Of the 22 prospective studies, twenty-one were cohort studies^6, 12, 28–46^ and one was a nested case-control study^47^.

**Table 1:** Study features of soy consumption and gastrointestinal cancer risk.

Reference	Location	Cancer type	Study years	Age	Cancer Size/Cohort Size	Intake measurements	Validity of FFQ	Soy consumption assessed	Cancer & death ascertainment
**Incidence**									
Umesawa^12^	Japan	Gastric cancer	1988–2009	40–79	787/40, 729	Self-administered FFQ	Yes	Miso soup	Population-based cancer registries; systematic review of death certificates
Hedelin^42^	Sweden	Colorectal cancer	1991–2010	30–49	Female: 206/48, 268	Self-administered FFQ	No	Isoflavonoids	Swedish cancer registry; total population register
Wada^6^	Japan	Gastric cancer	1992–2008	≥35	Male: 441/14, 219 Female: 237/16, 573	Self-administered FFQ	Yes	Miso soup, tofu (soy bean curd), deep-fried tofu, freeze-dried tofu, natto, houba-miso, soymilk, and boiled soy beans.	Regional population-based cancer registries; death certificate-only registration
Ko^41^	Korea	Gastric cancer	1993–2008	≥35	166/9724	Self-administered FFQ	No	Soybean/tofu, soybean pasta (miso soup)	Korean Central Cancer Registry; National Death Certificate databases
Kweon^46^	China	Gastric cancer	M: 2002–2006 F: 1996–2004	M:40–74 F: 40–70	Male: 324/61, 482 Female: 354/74, 941	In-person interview	Yes	Soy milk, Tofu, dry bean, fresh bean, bean sprout	Shanghai cancer registry; death certificate registries and confirmation through home visit.
Hara^40^	Japan	Gastric cancer	1995–2006	45–74	Male: 899/39,569 F: 350/45, 312	Self-administered FFQ	Yes	Miso soup, soymilk, tofu for miso soup, tofu for other dishes, yushidofu (predrained tofu), koyadofu (freeze-dried tofu), aburaage (deep-fried tofu), and natto (fermented soybeans)	Population-based cancer registries;
Yang^39^	China	Colorectal cancer	1997–2005	40–70	Female: 321/68, 412	In-person interview	Yes	Soy milk, tofu, fried tofu, dried or pressed tofu, fresh green soy beans, dry soy beans, soy sprouts, and other soy products	Population-based Shanghai Cancer Registry; Shanghai Municipal Center for Disease Control and Prevention
Wang^38^	USA	Colorectal cancer	1992–2005	≥45	Female: 3234/38, 408	Self-administered semi-quantitative FFQ	Yes	Tofu	Medical record review; death certificates
Butler^37^	Singapore Chinese	Colorectal cancer	1993–2005	45–74	Total: 961/61, 321	Self-administered Quantitative FFQ + Interview	Yes	Tofu in soups mixed dishes or alone, other tau kwa, foojook vegetarian meats, yong tau foo, other tau pok in soups	Population-based Singapore Cancer Registry; Singapore Registry of Births and Deaths
Akhter^36^	Japan	Colorectal cancer	1995–2004	45–74	Total: 886/83, 063	Self-administered FFQ	Yes	Miso soup, tofu (soybean curd) for miso soup, tofu (boiled or cold) for other dishes, yushidofu (predrained tofu), koyadofu or shimitofu (freeze-dried tofu), aburaage (deep-fried tofu), natto (fermented soybean), and soymilk (soybean as major ingredient).	Population-based cancer registries;
Oba^35^	Japan	Colon cancer	1992–2000	≥35	Male: 111/13,894 Female: 102/16, 327	Self-administered FFQ	Yes	Tofu, miso, soybeans, natto, soymilk, okara, dried tofu, fried tofu, deepfried tofu, and fried tofu with minced vegetables/seaweed	Regional population-based cancer registries; death certificate-only registration
Sauvaget^45^	Japan	Gastric cancer	1980–1999	34–98	1270/38, 576	Self-administered FFQ	Yes	Tofu (soybean curd), miso soup (soup made of a fermented and cooked soybeans paste)	Hospital records, physician notification and pathology records; Japanese family registration system
Galanis^28^	Hawaii, USA,	Gastric cancer	1975–1994	≥18	Male: 64/5, 610 Female: 44/6, 297	Interview FFQ	No	Miso soup	Hawaii Tumor Registry
Inoue 1996	Japan	Gastric cancer	1985–1995	NA	69/5, 373	Self-administered FFQ	No	Soybean-paste soup (miso soup)	Aichi prefectural cancer registry and death certificates
Ward^47^ (NCC)	European	Colorectal cancer	1993–2006	40–79	Male: 125/505 Female: 96/381	Self-administered healthy and lifestyle questionnaire	No	Isoflavones	Ease Anglia Cancer Registry
**Mortality**									
Iso^34^	Japan	Gastric cancer Colon cancer Rectal cancer	1988–2003	40–79	Male: 317/42,696 Female: 228/58, 494	Self-administered FFQ	Yes	Miso soup	Annually collected Death certifications with permission of Management and Coordination Agency of the Japanese Government
Kurosawa^33^	Japan	Gastric cancer	1989–1999	≥30	76/8, 035	Self-administered FFQ	No	Bean and bean products (cooked beans and bean curd and natto)	Population registries in the municipalities
Tokui^32^	Japan	Gastric cancer	1988–2003	40–79	859/110, 792	Self-administered FFQ	Yes	Bean curd, miso soup	Annually collected Death certifications with permission of Management and Coordination Agency of the Japanese Government
Khan^31^	Japan,	Gastric cancer Colorectal cancer	1984–2002	≥40	Male: 51/1, 524 F: 29/1,634	Staffs of the 45 health centers executed baseline survey and collected information	No	Tofu, miso soup, soybean curd, miso soup	By follow-up survey
Ngoan^30^	Japan,	Gastric cancer	1986–1994	≥15	Male: 77/5, 917 Female: 39/7,333	Self-administered FFQ	No	Tofu, soymilk, miso soup	Death forms from local health center with permission of the Management and Coordination Agency of the Japanese Government.
Nagata^29^	Japan	Gastric cancer	1992–1999	≥35	Male: 81/13, 880 Female: 40/16,424	Self-administered semi-quantitative FFQ	Yes	Tofu, miso, soybeans, natto, soymilk, okara, dried-tofu, deep-fried tofu, fried-tofu, fried tofu and minced vegetables/seaweed	Data from office of national vital statistics
Kato 1992	Japan	Gastric cancer	1985–1991	M: ≥40 F: ≥30	57/9,753	Self-administered FFQ	No	Miso soup	Examination of death certificates

FFQ: Food Frequency Questionnaire; NA: Not Available.

Among the 22 studies, Wada *et al*.^6^, Oba *et al*.^35^ and Nagata *et al*.^29^ reported on the gastric cancer incidence, colon cancer incidence and gastric cancer mortality, respectively, of the same study cohort. The studies by Kweon *et al*.^46^ and Yang *et al*.^39^ were based on the Shanghai health study cohort (China) and reported on the gastric cancer incidence and colorectal cancer incidence, respectively. Hara *et al*.^40^ and Akhter *et al*.^36^ reported the gastric cancer incidence and colorectal cancer incidence, respectively, of the Japan Public Health Center cohort. Umesawa *et al*.^12^, Iso *et al*.^34^ and Tokui *et al*.^32^ focused on the Japan Collaborative cohort. Although Iso *et al*.^34^ and Tokui *et al*.^32^ reported on the gastric cancer mortality of this cohort, Tokui *et al*.^32^ studied different exposure factors.

The included studies were published from 1990–2016. Among these studies, thirteen were conducted in Japan, two were conducted in the U.S., one was conducted in Korea, one was conducted in Sweden, one was conducted in China, one was conducted in Singapore and one was conducted at a multicenter in Europe. Thirteen of the included studies reported the outcomes of stomach cancer, seven studies reported the outcomes of colorectal cancer and two studies reported the outcomes of both stomach cancer and colorectal cancer.

All studies reported the association between soy intake and the incidence of mortality from gastrointestinal cancer. The Food Frequency Questionnaire (FFQ) was designed to assess the consumption of the specific food type used in each study independently. The reproducibility of the FFQs from thirteen of the studies was independently validated against previously reported studies. All studies clearly categorized several foods under the soy product group, except for those by Ward *et al*.^47^ and Hedelin *et al*.^42^, which only reported the intake of isoflavones (Table 2). Isoflavones are phytoestrogenic compounds that are abundant in soybeans. Eight studies discussed the association between the intake of isoflavones and risk of GI cancer. Miso soup was the most frequently reported soy product among the included studies, and thirteen studies evaluated the intake of miso soup. In the subgroup study, we conducted a meta-analysis of miso soup intake and GI cancer risk.

**Table 2:** The exposure type specific and gender specific risk estimates of GI cancer and soy consumption.

Reference	Cancer type	Exposure	RR, HR (95% CI)	Adjustments
**Incidence**				
Umesawa^12^	Gastric cancer	Miso soup		Age, sex, body mass index, ethanol intake, smoking status, family history of gastric cancer, walking time, educational status, and perceived mental stress.
		Both genders	1.66 (1.13–2.45)	
Hedelin^42^	Colorectal cancer	Isoflavone		Age, total energy intake, BMI, years of education, smoking status, physical activity, and dietary intake of processed meat, alcohol, saturated fat, vitamin D, vegetables, fruits, fish, and fiber, and individual, phytoestrogens, mutually adjusted for the phytoestrogen categories ligands, isoflavonoids, and coumestrol
		Female	1.06 (0.68, 1.65)	
Wada^6^	Gastric cancer	Soy product		Male: age, body mass index, physical activity score, smoking status, alcohol consumption, salt intake and education years
		Male	0.71 (0.53–0.96)	Female: age, body mass index, physical activity score, smoking status, alcohol consumption, salt intake, education years and menopausal status
		Female	0.58 (0.36–0.94)	
		Isoflavone		
		Male	0.81 (0.60–1.09)	
		Female	0.60 (0.37–0.98)	
Ko^41^	Gastric cancer	Soy product		Age, sex, cigarette smoking, body mass index, alcohol drinking, and area of residence
		Both genders	0.68 (0.38–1.21)	
		Male	0.77 (0.52–1.13)	
		Female	0.41 (0.22–0.78)	
		Miso soup		
		Both genders	2.01 (0.52–8.50)	
		Male	1.06 (0.93–1.21)	
		Female	1.10 (0.90–1.34)	
Kweon^46^	Gastric cancer	Soy product		Age, BMI, metabolic equivalents hours per week per year, chronic gastritis history, family gastric cancer history, born in urban Shanghai, family income, ever drink, ever smoke, and smoking amounts at baseline examinations as well as for median intakes of total calories, red meat, vegetables, sodium, fruit (excluding watermelon), and sex (for the models including both sexes, only).
		Both genders	0.72 (0.55, 0.95)	
		Male	0.64 (0.42, 0.99)	
		Female	0.82 (0.57, 1.17)	
Hara^40^	Gastric cancer	Soy product		Age, public center area, BMI, smoking status, ethanol intake, family history of gastric cancer, vegetable intake, fruit intake, fish intake, salt intake, and total energy intake.
		Male	1.02 (0.82, 1.25)	
		Female	0.99 (0.71, 1.38)	
		Isoflavone		
		Male	1.00 (0.81, 1.24)	
		Female	1.07 (0.77, 1.50)	
		Miso soup		
		Male	1.17 (0.94, 1.47)	
		Female	0.71 (0.50, 1.01)	
Yang^39^	Colorectal cancer	Soy product		Age, education, household income, physical activity, BMI, menopausal status, family history of colorectal cancer, total calorie intake, and average intakes of fruit, vegetables, red meat, non-soy calcium, non-soy fiber, and non-soy folic acid and was stratified by birth year.
		Female	0.67 (0.49, 0.90)	
		Isoflavones		
		Female	0.76 (0.56, 1.01)	
Wang^38^	Colorectal cancer	Soy product		Age; race; total energy intake; randomized treatment assignment; smoking; alcohol use, physical activity; postmenopausal status; hormone replacement therapy use; multivitamin use; BMI; family history of colorectal cancer, ovary cancer, and breast cancer; and intake of fruit and vegetables, fiber, folate, and saturated fat.
		Female	0.54 (0.20,1.46)	
Butler^37^	Colorectal cancer	Soy product		Age, sex, dialect group, interview year, diabetes at baseline, smoking history, alcohol intake, education, any weekly physical activity, first-degree relative diagnosed with colorectal cancer, and total daily energy intake.
		Both genders	0.95 (0.78–1.16)	
		Isoflavones		
		Both genders	0.95 (0.79–1.13)	
Akhter^36^	Colorectal cancer	Soy product		Age; public health center area; history of diabetes mellitus; body mass index; leisure time physical activity; cigarette smoking; alcohol drinking; and intake of vitamin D, dairy products, meat, vegetable, fruit, and fish. Also adjusted for menopausal status and current use of female hormones in women only.
		Male	0.89 (0.68–1.17)	
		Female	1.04 (0.76–1.42)	
		Isoflavones		
		Male	0.89 (0.67–1.17)	
		Female	1.07 (0.78–1.47)	
		Miso soup		
		Male	0.88 (0.64–1.10)	
		Female	1.03 (0.75–1.43)	
Oba^35^	Colon cancer	Soy product		Age, height, alcohol intake, smoking status, BMI, physical exercise, coffee intake, and use of hormone replacement therapy (women only).
		Male	1.24 (0.77–2.00)	
		Female	0.56 (0.34–0.92)	
		Isoflavones		
		Male	1.47 (0.90–2.40)	
		Female	0.73 (0.44–1.18)	
Sauvaget^45^	Gastric cancer	Soy product		Sex-specific age, sex, city, radiation dose, sex-specific smoking habits, and education level
		Both genders	1.01 (0.85–1.20)	
		Miso Soup		
		Both genders	1.01 (0.88–1.16)	
Galanis^28^	Gastric cancer	Miso Soup		Age, years of education, Japanese place of birth, and gender (In combined analyses). Analyses among men were also adjusted for cigarette smoking and alcohol intake status
		Both genders	1.2 (0.8–1.8)	
		Male	1.2 (0.7–2.0)	
		Female	1.3 (0.7–2.4)	
Inoue 1996	Gastric cancer	Miso Soup		Age and sex
		Both genders	3.62 (0.79–16.70)	
Ward^47^	Colorectal cancer	Isoflavones		Age, height, weight, family history of colorectal cancer, smoking status, aspirin use, physical activity, and average daily intake of fat, energy, calcium, fiber, alcohol, and red and processed meats.
		Male	1.12 (0.88, 1.42)	
		Female	1.19 (0.92, 1.54)	
**Mortality**				
Iso^34^	Gastric cancer	Miso soup		Age
		Male	0.96 (0.77–1.20)	
		Female	1.18 (0.89–1.58)	
	Colon cancer	Miso soup		
		Male	0.87 (0.58–1.28)	
		Female	0.84 (0.58–1.23)	
	Rectal cancer	Miso soup		
		Male	0.75 (0.48–1.18)	
		Female	1.02 (0.56–1.85)	
Kurosawa^33^	Gastric cancer	Soy product		Age, sex, highly salted food, green and yellow vegetables, beans and bean products, mountain herbs, fruits, and the smoking habit
		All	0.88 (0.31–2.56)	
Tokui^32^	Gastric cancer	Soy product		Age
		Male	1.07 (0.73–1.58)	
		Female	1.41 (0.75–2.64)	
Khan^31^	Gastric cancer	Soy product		Age, health status, health education, health screening and smoking;
		Male	3.6 (0.5–26.0)	Male: age and smoking
		Female	1.1 (0.1–8.5)	
		Miso soup		
		Male	0.2 (0.1–0.8)	
	Colorectal cancer	Soy product		
		Male	1.5 (0.2–11.2)	
		Female	0.9 (0.1–6.9)	
Ngoan^30^	Gastric cancer	Soy product		Both genders: age, sex, smoking, and other dietary factors (processed meat, liver, cooking oil, sui mono, and pickled food),
		Both genders	0.4 (0.2–0.9)	Gender specific: age
		Male	0.9 (0.4–1.8)	
		Female	0.8 (0.3–2.2)	
		Miso soup		
		Both genders	1.7 (0.6–4.5)	
Nagata^29^	Gastric cancer	Soy product		Male: age, total energy, smoking status (current, former, and never-smokers) and body mass index at age about 21 years;
		Male	0.48 (0.27–0.83)	Female: age, total energy, marital status, age at menarche, and body mass index at age about 21 years.
		Female	0.49 (0.21–1.12)	
Kato 1992	Gastric cancer	Miso soup		Age and sex
		Both genders	1.04 (0.48–2.25)	

RR: Relative Risk; HR: Hazard Ratio; CI: Confidence Intervals; BMI: Body Mass Index.

The data collection method that was used for the three studies was an in-person interview, while the remainder of the 19 studies used a self-administered FFQ.

Three studies adjusted for the confounding factors of age and sex, while the remaining 19 studies applied multiple adjustments. The exposure type and gender-specific risk estimates of GI cancer and the adjustments for confounding factors are shown in Table 2.

### Quantitative synthesis

#### Soy consumption and GI cancer incidence

In our meta-analysis, the intake of mixed soy types had no cancer site-specific or gender-specific association with GI cancer incidence.

**Figure 2:**
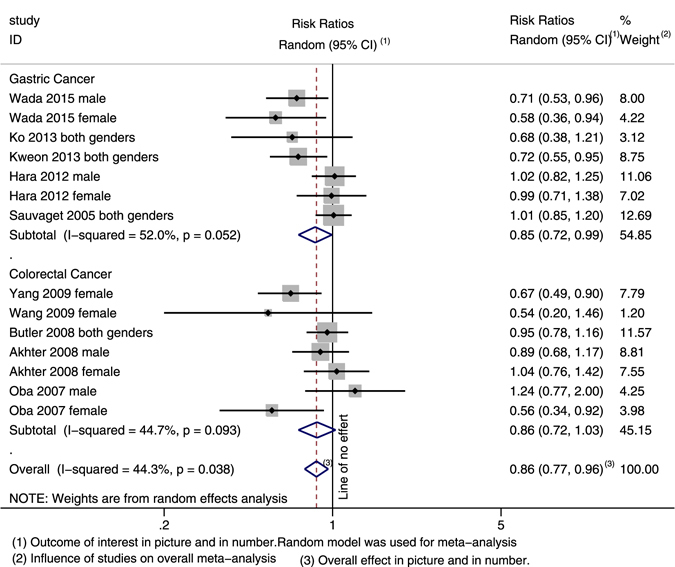
Forest plot and summary risk estimates for both genders of the association between soy product intake and incidence of GI cancer.

**Figure 3:**
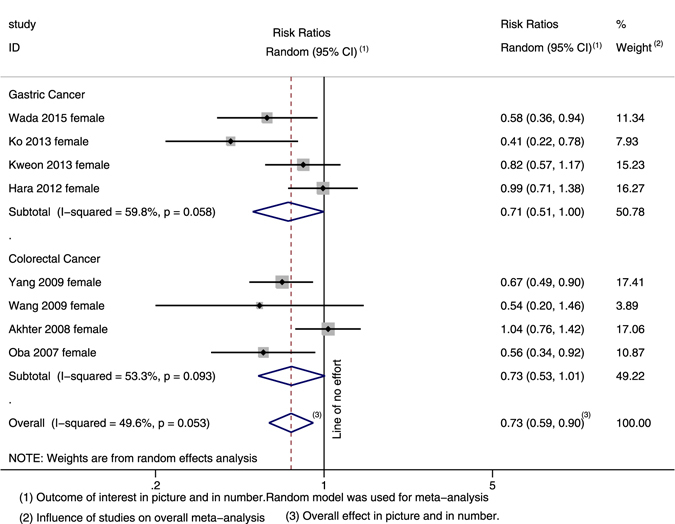
Forest plot and summary risk estimates for females of the association between soy product intake and incidence of GI cancer.

Ten studies focused on the association between soy product intake and incidence of GI cancer. The highest versus the lowest categories of soy product consumption were inversely associated with the incidence of overall GI cancer (0.857; 95% CI: 0.766, 0.959; Heterogeneity: *I²* = 44.3%) and the gastric cancer subgroup (0.847; 95% CI: 0.722, 0.994; Heterogeneity: *I²* = 52.0%) but not the colorectal cancer subgroup (0.862; 95% CI: 0.722, 1.030; Heterogeneity: *I²* = 44.3%) (Fig. 2). After stratifying according to gender, we found an inverse association between soy product intake and the incidence of GI cancer for females but not for males. Eight studies reported on the outcomes for females. The pooled RR was 0.730 (95% CI: 0.591, 0.903; Heterogeneity: *I²* = 49.6%) for overall GI cancer, 0.711 (95% CI: 0.506, 0.999; Heterogeneity: *I²* = 59.8%) for gastric cancer and 0.734 (95% CI: 0.533, 1.010; Heterogeneity: *I²* = 53.3%) for colorectal cancer (Fig. 3). Among the males, no association was observed between soy product intake and the incidence of overall GI cancer, incidence of gastric cancer, or incidence of colorectal cancer.

Eight studies reported the association between isoflavone intake and the incidence of GI cancer. The highest versus the lowest categories of isoflavone intake had no cancer site-specific or gender-specific associations with GI cancer.

Seven studies reported the association between miso soup intake and the incidence of GI cancer. No gender-specific or cancer site-specific associations were detected between miso soup intake and GI cancer incidence.

#### Soy consumption and GI cancer mortality

The estimated summary risk for the highest versus the lowest categories of soy consumption showed no association with the mortality of overall GI cancer, mortality of gastric cancer, or mortality of colorectal cancer. After stratifying according to gender, no association was observed for females or males.

In the subgroup analysis, we stratified by exposure, and no association was detected for soy product intake or miso soup intake.

Detailed results of the subgroup analysis are summarized in Table 3.

**Table 3:** Pooled risk estimates between lowest categories compared with highest categories of soy consumption and gastrointestinal cancer risk.

Exposure	Gender difference	Gastrointestinal	Gastric	Colorectal	I2	Begg’s test	Egger’s test
**Incidence**							
Mixed exposure	Both genders	0.941 (0.841, 1.052)	0.939 (0.782, 1.127)	0.947 (0.820, 1.094)	56.4%	0.581	0.682
	Male	0.922 (0.791, 1.074)	0.843 (0.680, 1.046)	1.039 (0.871, 1.240)	43.8%	0.902	0.648
	Female	0.828 (0.680, 1.009)	0.778 (0.562, 1.076)	0.865 (0.662, 1.129)	59.8%	0.213	0.117
Soy product	Both genders	0.857 (0.766, 0.959)*	0.847 (0.722, 0.994)*	0.862 (0.722, 1.030)	44.3%	0.101	0.044*
	Male	0.862 (0.726, 1.024)	0.804 (0.640, 1.010)	0.965 (0.762, 1.222)	40.9%	0.707	0.532
	Female	0.730 (0.591, 0.903)*	0.711 (0.506, 0.999)*	0.734 (0.533, 1.010)	49.6%	0.108	0.075
Isoflavone	Both genders	0.973 (0.899, 1.054)	0.897 (0.733, 1.097)	0.997 (0.907, 1.096)	29.2%	0.760	0.594
	Male	0.996 (0.882, 1.124)	0.931 (0.783, 1.018)	1.078 (0.851, 1.366)	31.2%	1.000	0.609
	Female	0.936 (0.781, 1.123)	0.824 (0.469, 1.449)	0.967 (0.791, 1.181)	44.9%	0.133	0.179
Miso soup	Both genders	1.064 (0.956, 1.183)	1.094 (0.966, 1.238)	0.939 (0.763, 1.156)	41.1%	0.213	0.266
	Male	1.059 (0.956, 1.173)	1.092 (0.977, 1.220)	0.880 (0.671, 1.154)	0.0%	1.000	0.984
	Female	0.933 (0.798, 1.235)	0.977 (0.701, 1.362)	1.030 (0.746, 1.422)	42.6%	0.734	0.826
**Mortality**							
Mixed exposure	Both genders	0.926 (0.824, 1.041)	0.898 (0.707, 1.142)	0.854 (0.689, 1.041)	23.1%	0.902	0.636
	Male	0.897 (0.771, 1.043)	0.889 (0.648, 1.218)	0.826 (0.616, 1.108)	19.6%	1.000	0.16
	Female	1.017 (0.840, 1.231)	1.100 (0.865, 1.399)	0.888 (0.648, 1.216)	0.0%	0.902	0.453
Soy product	Both genders	0.831 (0.665–1.038)	0.796 (0.573, 1.106)	1.177 (0.274, 5.061)	30.2%	0.680	0.825
	Male	0.883 (0.541, 1.444)	0.864 (0.503, 1.486)	1.500 (0.200, 11.225)	48.0%	1.000	0.628
	Female	0.932 (0.606, 1.434)	0.933 (0.601, 1.449)	0.900 (0.108, 7.476)	1.0%	0.806	0.731
Miso soup	Both genders	0.917 (0.753, 1.118)	0.942 (0.645, 1.376)	0.848 (0.683, 1.054)	42.3%	0.754	0.372
	Male	0.752 (0.520, 1.089)	0.477 (0.104, 2.200)	0.815 (0.606, 1.098)	66.1%	0.089	0.060
	Female	1.038 (0.839, 1.285)	1.180 (0.886, 1.572)	0.888 (0.646, 1.220)	0.0%	1.000	0.712

^*^Statistically significant (P < 0.05).

#### Publication bias and sensitivity analysis

The results of the Begg–Mazumdar test and Egger’s test indicated no evidence of a substantial publication bias for most of the analyses, except for the analysis of soy product consumption and the incidence of GI cancer for both genders. Although this analysis showed a publication bias under Egger’s test, it did not show one under the Begg–Mazumdar or funnel test. We strictly followed our inclusion criteria, and therefore, we determined that the results did not suggest any publication bias.

**Figure 4:**
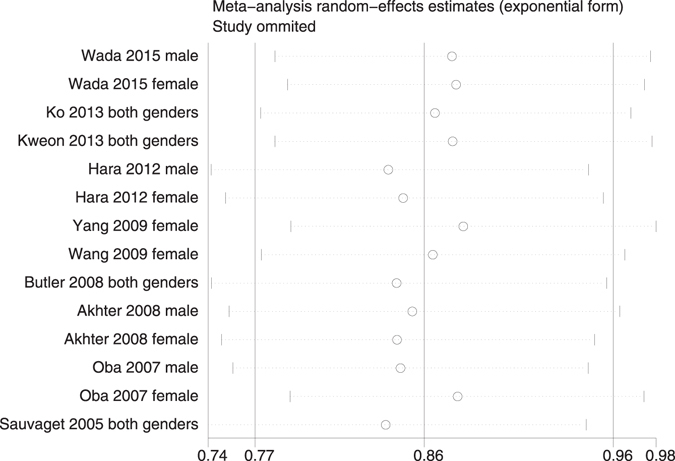
Sensitivity analysis for both genders of soy product intake and incidence of GI cancer.

**Figure 5:**
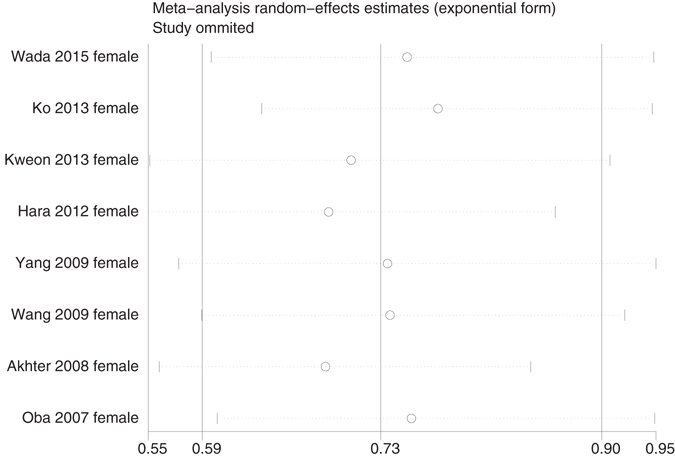
Sensitivity analysis for females of soy product intake and incidence of GI cancer.

We applied a sensitivity analysis on our positive meta-analysis results. The overall pooled estimate did not substantially vary with the exclusion of any single study (Figs 4 and 5).

## Discussion

We systematically reviewed the existing literature from three main databases and identified 22 prospective epidemiological studies that assessed the association between soy consumption and GI cancer risk. The findings showed that there was no association between soy consumption and GI cancer risk. Cancer site-specific and soy subtype-specific subgroup analyses revealed that the highest versus the lowest categories of soy product consumption were inversely associated with the incidence of overall GI cancer and the gastric cancer subgroup, but not the colorectal cancer subgroup. A gender-specific analysis showed that this protective effect that the soy product has on the incidences of GI cancer and gastric cancer was only observed in females.

Our results did not find any association between soy consumption and colorectal cancer risk, which was consistent with some previous meta-analyses, including Yan *et al*.^22^ and Jin *et al*.^48^. However, Tse *et al*.^15^, Yu *et al*.^26^ and Zhu *et al*.^49^ reported that soy consumption had an inverse association with CRC. Although the previous studies were inconsistent, our study included the newly reported articles by Umesawa *et al*.^12^ and Hedelin *et al*.^42^, both of which reported no association between GI cancer risk and soy consumption. Woo *et al*. (2013) performed a meta-analysis of the risks of gastric and colorectal cancer with flavonoids intake^14^. The inclusion of this study showed no association between colorectal cancer risk and flavonoids intake when case-control designed studies were excluded, while a significant inverse association was detected when case-control designed studies were included. Our meta-analysis included only prospective studies, which minimized the recall bias and selection bias from case-control studies, while most retrospective studies reported a significant inverse association. Thus, our most updated and prospective studies included only a meta-analysis, which was more reliable.

Several mechanisms may account for the inverse association between soy product consumption and the incidence of gastric cancer. Two of the major soy isoflavones are genistein and daidzein, which have anti-inflammatory and antioxidative effects^50^. Genistein is known to inhibit the growth of H. pylori^51^ and the activation of the nuclear factor-kappaB (NF-κB) signaling pathway. The classical activation pathway of NF-κB signaling has been identified in regulating inflammation-associated gastrointestinal tract malignancies^52–54^. Genistein also reduced the growth and proliferation of gastric cancer cells by cell cycle arrest and the Akt signaling pathway, which increased apoptosis and inhibited angiogenesis^55–57^.

Interestingly, this protective effect was only found for soy product consumption but not for the mixed exposure. Of all of the included studies, seven studies reported the association between soy product consumption and the incidence of gastric cancer^6, 40, 41, 45, 46^. Wada *et al*.^6^ and Hara *et al*.^40^ reported this association in females and males, respectively. Thus, we considered them to be two independent studies. Those seven studies that had a clear statement on the measurement of the intake of the mixed types of soybean products are shown in Table 1. However, there were three studies^12, 28, 44^ that reported the relationship between miso soup consumption and the incidence of gastric cancer. When we combined those three studies with the previous seven studies that included a mixed exposure, the above-mentioned protective effect was not observed. Miso soup is a traditional Japanese food with high salt that is made from fermented soybeans^58^. The fermented soy foods contain N-nitroso compounds. High concentrations of sodium in the diet were reported to enhance the carcinogenicity of N-nitroso compounds and H. pylori infection, as well as weaken the protective effect of the mucous barrier^12, 59, 60^.

In our study, the beneficial effect of soy consumption was found among the female population but not among the male population. Chandanos *et al*. reported that women with a longer fertility life and those who are on hormone replacement therapy seem to have a decreased risk of gastric cancer, and men who have been treated with estrogen for prostate cancer also have a decreased risk^61^. The mechanism for this decrease in risk remains unknown. Isoflavones have a similar structure to 17β-estradiol and act as estrogen agonists or antagonists in environments of different estrogen levels, which may contribute to the different beneficial effects of soy consumption in females and males^62^.

Moderate heterogeneity was found from some of our results. First, while every study adjusted for age and gender in the calculation of risk estimates, not every included study has been adjusted for total energy intake and body mass index, which are confounding factors^63^. Second, the effects that soy intake has on GI cancer risk might differ among different preparations or fermentations of soy foods. Three included studies adjusted and analyzed fermented and non-fermented soy food^6, 40, 46^. The high intake of non-fermented soy food was more likely to be inversely associated with gastric cancer risk^6^. A higher salt intake increased the risk of GI cancer, and miso soup, one of the soy subtypes, was considered a high salt food^12, 64^. Third, the data gathering methods that were used might also contribute to the heterogeneity. Four studies relied on a personal interview, while the remaining studies came from the self-reported Food Frequency Questionnaires (FFQ)^28, 31, 39, 46^. The participants may have different understandings of the questionnaire by different methods. Fourth, thirteen studies used a validated FFQ mixed with nine non-validated FFQs. The validated FFQ listed various types of soy foods, leading to precise estimates of soy or isoflavone intake. Fifth, we have pooled cohort studies and a nested case-control study with different estimates of OR, RR and HR. HR and OR were considered to be approximations of RR because CRC is a rare outcome in humans. We used a random effects method to determine when the heterogeneity (*I* ^*2*^) was larger than 40% to enhance the credibility of the results.

Our meta-analysis has several strengths. First, our study was based on only prospective studies, which enabled us to minimize the food exposure recall bias and selection bias. To our knowledge, this is the first time that the association between both GI cancer incidence and mortality with soy intake from prospective studies has been summarized. Most previous meta-analyses collected both retrospective and prospective studies. Woo *et al*. (2013) reported that a case-control design created a significant association between the flavonoid subclasses and cancer risk, while cohort studies did not observe this association^14^. Second, all included studies strictly followed our inclusion criteria, which made our results more stable. Third, our sample size is an important strength, as we included a total of 12,901 cancer cases from a total of 965,466 participants. Combining a large number of participants renders us sufficient power to detect potential, modest associations. Fourth, according to our sensitivity analysis, the inverse association did not vary with the exclusion of any single study.

Similar to all other meta-analyses, our study has some limitations. First, moderate heterogeneity was observed from some of our results. We have discussed the reasons above; however, the sensitivity analysis showed that our inverse association was stable and reliable. Second, the included studies were reported from different countries and populations and the measurement of soy intake and soy type varied among them.

In summary, no association was found between soy consumption and GI cancer incidence or mortality. A higher intake of soy product is associated with the decreased risk of overall GI cancer and gastric cancer, but not colorectal cancer. This protective effect was observed in females but not in males.

## Methods

### Search strategy

We systematically searched three databases, PubMed, ISI web of science and EMBASE, for studies that were published in any language (up until December 7, 2016). We combined the key words of the three following items: terms for outcome (colorectal cancer, gastric cancer, or gastrointestinal cancer), terms for exposure (soy product or isoflavone), and terms for epidemiology (cohort, prospective, or observational study).

According to the key words of the medical subject headings (MeSH), we searched the following MeSH: colorectal cancer, colorectal carcinoma, colorectal neoplasm(s), colorectal tumor(s), colon cancer, colon carcinoma, colon neoplasm(s), colon tumor(s), colonic cancer, colonic carcinoma, colonic neoplasm(s), colonic tumor(s), rectal cancer, rectal carcinoma, rectal neoplasm(s), rectal tumor(s), rectum cancer, rectum carcinoma, rectum neoplasm(s), rectum tumor(s), stomach cancer, stomach carcinoma, stomach neoplasm(s), stomach tumor(s), gastric cancer, gastric carcinoma, gastric neoplasm(s), gastric tumor(s), gastrointestinal cancer, gastrointestinal carcinoma, gastrointestinal neoplasm(s), gastrointestinal tumor(s), soy, tofu, miso, soybean, soymilk, natto, isoflavone, coumestrol, genistein, pterocarpans, daidzein, cohort, prospective, and observational study. This search was restricted to studies that used human participants.

In addition, we reviewed the reference lists of all of the eligible studies to identify more potential studies.

### Study selection

The following inclusion criteria were applied in the screening of articles: (1) original reported data that evaluated the association between soy consumption and GI cancer incidence or mortality, (2) studies with a prospective study design, (3) studies that used risk point estimates, e.g., odds ratio (OR), relative risk (RR) or hazard ratio (HR) estimates with 95% confidence intervals (CIs), and (4) studies with population-based control samples. We did not include the studies that reported the associations between the serum concentrations of isoflavones and GI risk. When there were multiple published reports from the same study population, the most recent or the most informative report was selected for analysis.

### Data extraction

The extracted data that were used included the first author’s name, year of publication, participants’ ages, study name, location, sample size, cancer type, study period, method used for the food intake measurements, validity of FFQ, method used in the cancer and/or death ascertainment, exposure items, soy consumption type, the risk estimates or data used to calculate the risk estimates, 95% CIs and adjustments for potential confounding effects. When more than one adjusted ratio was reported, the ratio with the most adjustment variables was chosen.

### Credibility of meta-analysis results

We performed this meta-analysis under the guidance of Preferred Reporting Items for Systematic reviews and Meta-Analyses (PRISMA)^65^ and Meta-analysis of Observational Studies in Epidemiology (MOOSE)^66^. All enrolled studies were in strict compliance with well-designed inclusion criteria and exclusion criteria. To protect from bias, there was no change of results when any of the studies were excluded by the sensitivity analysis. Two observers independently evaluated the quality and eligibility of the included studies.

### Statistical analysis

We extracted the association between soy consumption and GI cancer incidence or mortality by the ORs, RRs or HRs that were reported in the included studies. Soy type was defined as being one of three subgroups: soy product, isoflavone or miso soup. When more than one adjusted ratio was reported, the ratio with the most adjustment variables was chosen. ORs, RRs or HRs and 95% CIs were estimated based on the most adjusted variables for the highest versus the lowest soy consumption. In situations where the incidence was low, the odds ratio approximates the relative risk and hazard ratio. Therefore, for studies of GI cancer (a rare event), it is acceptable to compare the OR, RR and HR estimates^67–69^. The outcomes are presented as a forest plot with the 95% CIs.

We used *I* ^*2*^ and Cochrane Q statistics, which are quantitative measures of inconsistency among studies, to test for possible heterogeneity across the studies^70^. When *I* ^*2*^ was from 0% to 40% and had a P > 0.10, the heterogeneity might not be important. If the meta-analysis has no heterogeneity, a fixed-effects model with the Mantel–Haeszel method^71^ would be used to combine the individual studies. Otherwise, the random-effects method^72^ was used for pooling.

To estimate multiple modification effects, cancer site-specific, gender-specific and soy type-specific analyses were performed. Additionally, we did a single study sensitivity analysis for each of the statistically significant results. Sensitivity analyses were conducted by excluding each study, in turn, to evaluate the stability of the results.

The Egger’s regression test^73^ and Begg–Mazumdar test^74^ were used to assess for publication bias. P < 0.05 was considered to be a statistically significant publication bias.

All reported P-values were two-sided. All statistical analyses were performed using STATA (version 11.0; Stata-Corp, College Station, TX).

## References

[1] Dong J-Y, Qin L-Q (2011). Soy isoflavones consumption and risk of breast cancer incidence or recurrence: a meta-analysis of prospective studies. Breast cancer research and treatment.

[2] Nagata C (2010). Factors to consider in the association between soy isoflavone intake and breast cancer risk. Journal of epidemiology.

[3] Caan BJ (2011). Soy food consumption and breast cancer prognosis. Cancer Epidemiology Biomarkers & Prevention.

[4] Budhathoki S (2011). Soy food and isoflavone intake and colorectal cancer risk: the Fukuoka Colorectal Cancer Study. Scandinavian journal of gastroenterology.

[5] Hsu A, Bray TM, Helferich WG, Doerge DR, Ho E (2010). Differential effects of whole soy extract and soy isoflavones on apoptosis in prostate cancer cells. Experimental biology and medicine.

[6] Wada K (2015). Soy isoflavone intake and stomach cancer risk in Japan: From the Takayama study. International journal of cancer. Journal international du cancer.

[7] Bingham SA (2003). Dietary fibre in food and protection against colorectal cancer in the European Prospective Investigation into Cancer and Nutrition (EPIC): an observational study. The Lancet.

[8] Ko KP (2010). Isoflavones from phytoestrogens and gastric cancer risk: a nested case-control study within the Korean Multicenter Cancer Cohort. Cancer epidemiology, biomarkers & prevention: a publication of the American Association for Cancer Research, cosponsored by the American Society of Preventive Oncology.

[9] Ross JA, Kasum CM (2002). Dietary flavonoids: bioavailability, metabolic effects, and safety. Annual review of nutrition.

[10] Neuhouser ML (2004). Review: dietary flavonoids and cancer risk: evidence from human population studies. Nutrition and cancer.

[11] Arts IC, Hollman PC (2005). Polyphenols and disease risk in epidemiologic studies. The American journal of clinical nutrition.

[12] Umesawa M (2016). Salty Food Preference and Intake and Risk of Gastric Cancer: The JACC Study. Journal of Epidemiology.

[13] Tong, X., Li, W. & Qin, L. Q. [Meta-analysis of the relationship between soybean product consumption and gastric cancer]. Zhonghua yu fang yi xue za zhi [Chinese journal of preventive medicine] 44, 215–220 (2010). 20450742

[14] Woo HD, Kim J (2013). Dietary flavonoid intake and risk of stomach and colorectal cancer. World journal of gastroenterology.

[15] Tse G, Eslick GD (2016). Soy and isoflavone consumption and risk of gastrointestinal cancer: a systematic review and meta-analysis. European journal of nutrition.

[16] Chyou PH, Nomura AM, Stemmermann GN (1995). Diet, alcohol, smoking and cancer of the upper aerodigestive tract: a prospective study among Hawaii Japanese men. International journal of cancer. Journal international du cancer.

[17] Cutler GJ (2008). Dietary flavonoid intake and risk of cancer in postmenopausal women: the Iowa Women’s Health Study. International journal of cancer. Journal international du cancer.

[18] Shu XO (2015). Cohort Profile: The Shanghai Men’s Health Study. International journal of epidemiology.

[19] Zamora-Ros R (2010). Estimation of Dietary Sources and Flavonoid Intake in a Spanish Adult Population (EPIC-Spain). Journal of the American Dietetic Association.

[20] Ito L. S., e. a. Dietary Factors and the Risk of Gastric Cancer Among Japanese Women: a Comparison Between the Differentiated and Non-Differentiated Subtypes. AEP Vol. 13, No. 1 January 2003, 2024–2031 (2003). 10.1016/s1047-2797(02)00269-7 12547482

[21] Nomura A, J. S. G, Stemmermann GN, Severson RK (1990). A Prospective Study of Stomach Cancer and Its Relation to Diet, Cigarettes, and Alcohol Consumption. Cancer Research.

[22] Yan L, Spitznagel EL, Bosland MC (2010). Soy consumption and colorectal cancer risk in humans: a meta-analysis. Cancer epidemiology, biomarkers & prevention: a publication of the American Association for Cancer Research, cosponsored by the American Society of Preventive Oncology.

[23] Hara A (2013). Plasma Isoflavone Concentrations Are Not Associated with Gastric Cancer Risk among Japanese Men and Women. Journal of Nutrition.

[24] Golpour S, Rafie N, Safavi SM, Miraghajani M (2015). Dietary isoflavones and gastric cancer: A brief review of current studies. Journal of research in medical sciences: the official journal of Isfahan University of Medical Sciences.

[25] Wang Y (2015). Fruit and vegetable consumption and risk of lung cancer: A dose–response meta-analysis of prospective cohort studies. Lung cancer (Amsterdam, Netherlands).

[26] Yu Y, Jing X, Li H, Zhao X, Wang D (2016). Soy isoflavone consumption and colorectal cancer risk: a systematic review and meta-analysis. Scientific reports.

[27] Thorning TK (2016). Milk and dairy products: good or bad for human health? An assessment of the totality of scientific evidence. Food Nutr Res.

[28] Galanis, D. J. Intakes of selected foods and beverages and the incidence of gastric cancer among the Japanese residents of Hawaii: a prospective study (1998). 10.1093/ije/27.2.173 9602395

[29] Nagata C, Takatsuka N, Kawakami N, Shimizu H (2002). A prospective cohort study of soy product intake and stomach cancer death. British journal of cancer.

[30] Ngoan LT, Mizoue T, Fujino Y, Tokui N, Yoshimura T (2002). Dietary factors and stomach cancer mortality. British journal of cancer.

[31] Khan MM (2004). Dietary habits and cancer mortality among middle aged and older Japanese living in hokkaido, Japan by cancer site and sex. Asian Pacific J Cancer Prev.

[32] Tokui N (2005). Dietary habits and stomach cancer risk in the JACC Study. Journal of Epidemiology.

[33] Kurosawa M, Kikuchi S, Xu J, Inaba Y (2006). Highly salted food and mountain herbs elevate the risk for stomach cancer death in a rural area of Japan. J Gastroenterol Hepatol.

[34] Iso H, Kubota Y (2007). Nutrition and disease in the Japan Collaborative Cohort Study for Evaluation of Cancer (JACC). Asian Pacific J Cancer Prev.

[35] Oba S (2007). Soy product consumption and the risk of colon cancer: a prospective study in Takayama, Japan. Nutr Cancer.

[36] Akhter M (2008). Dietary soy and isoflavone intake and risk of colorectal cancer in the Japan public health center-based prospective study. Cancer Epidemiology Biomarkers & Prevention.

[37] Butler LM, Wang R, Koh WP, Yu MC (2008). Prospective study of dietary patterns and colorectal cancer among Singapore Chinese. British journal of cancer.

[38] Wang L (2009). Dietary intake of selected flavonols, flavones, and flavonoid-rich foods and risk of cancer in middle-aged and older women. Am J Clin Nutr.

[39] Yang G (2009). Prospective cohort study of soy food intake and colorectal cancer risk in women. American Journal of Clinical Nutrition.

[40] Hara A (2012). Isoflavone intake and risk of gastric cancer: a population-based prospective cohort study in Japan. American Journal of Clinical Nutrition.

[41] Ko K-P (2013). Intake of Soy Products and Other Foods and Gastric Cancer Risk: A Prospective Study. Journal of Epidemiology.

[42] Hedelin M, Lof M, Sandin S, Adami HO, Weiderpass E (2016). Prospective Study of Dietary Phytoestrogen Intake and the Risk of Colorectal Cancer. Nutr Cancer.

[43] I., K., S., T. & K., M. A prospective study of stomach cancer among a rural Japanese Population: a 6- year survey Japanese journal of cancer research: Gann 83, 568–575 (1992). 10.1111/j.1349-7006.1992.tb00127.x PMC5918886 1644660

[44] Tajima MIK (1996). Protective factor against progression from atrophic gastritis to gastric cancer-data from a cohort study in Japan. International journal of cancer. Journal international du cancer.

[45] Sauvaget C (2005). Lifestyle factors, radiation and gastric cancer in atomic-bomb survivors (Japan). Cancer causes & control: CCC.

[46] Kweon SS (2013). Intake of specific nonfermented soy foods may be inversely associated with risk of distal gastric cancer in a Chinese population. The Journal of nutrition.

[47] Ward HA (2010). Breast, colorectal, and prostate cancer risk in the European Prospective Investigation into Cancer and Nutrition-Norfolk in relation to phytoestrogen intake derived from an improved database. American Journal of Clinical Nutrition.

[48] Jin H, Leng Q, Li C (2012). Dietary flavonoid for preventing colorectal neoplasms. The Cochrane database of systematic reviews.

[49] Zhu B, Sun Y, Qi L, Zhong R, Miao X (2015). Dietary legume consumption reduces risk of colorectal cancer: evidence from a meta-analysis of cohort studies. Scientific reports.

[50] T. S, M. K (2008). Soy isoflavones and immunity. The journal of medical investigation: JMI.

[51] Verdrengh M, Collins LV, Bergin P, Tarkowski A (2004). Phytoestrogen genistein as an anti-staphylococcal agent. Microbes Infect.

[52] Merga YJ (2016). Importance of the alternative NF-kappaB activation pathway in inflammation-associated gastrointestinal carcinogenesis. American journal of physiology. Gastrointestinal and liver physiology.

[53] Maeda S, Omata M (2008). Inflammation and cancer: role of nuclear factor-kappaB activation. Cancer science.

[54] Sarkar FH, Li Y (2002). Mechanisms of cancer chemoprevention by soy isoflavone genistein. Cancer metastasis reviews.

[55] Yao H, Xu W, Shi X, Zhang Z (2011). Dietary flavonoids as cancer prevention agents. Journal of environmental science and health. Part C, Environmental carcinogenesis & ecotoxicology reviews.

[56] Banerjee S, Li Y, Wang Z, Sarkar FH (2008). Multi-targeted therapy of cancer by genistein. Cancer letters.

[57] Yang ZP (2015). Equol inhibits proliferation of human gastric carcinoma cells via modulating Akt pathway. World journal of gastroenterology.

[58] Ito K, Hirooka Y, Sunagawa K (2014). Miso (Japanese soybean paste) soup attenuates salt-induced sympathoexcitation and left ventricular dysfunction in mice with chronic pressure overload. Fukuoka igaku zasshi = Hukuoka acta medica.

[59] Kim J (2011). Fermented and non-fermented soy food consumption and gastric cancer in Japanese and Korean populations: a meta-analysis of observational studies. Cancer science.

[60] Nozaki K (2002). Synergistic promoting effects of Helicobacter pylori infection and high-salt diet on gastric carcinogenesis in Mongolian gerbils. Japanese journal of cancer research: Gann.

[61] Chandanos E, Lagergren J (2008). Oestrogen and the enigmatic male predominance of gastric cancer. European journal of cancer (Oxford, England: 1990).

[62] Hwang CS (2006). Isoflavone metabolites and their *in vitro* dual functions: they can act as an estrogenic agonist or antagonist depending on the estrogen concentration. The Journal of steroid biochemistry and molecular biology.

[63] Willett, W. C., Howe, G. R. & Kushi, L. H. Adjustment for total energy intake in epidemiologic studies. Am J Clin Nutr 65, 1220S–1228S; discussion 1229S–1231S (1997). 10.1093/ajcn/65.4.1220S 9094926

[64] Kim J, Park S, Nam BH (2010). Gastric cancer and salt preference: a population-based cohort study in Korea. Am J Clin Nutr.

[65] Moher D, Liberati A, Tetzlaff J, Altman DG (2010). Preferred reporting items for systematic reviews and meta-analyses: the PRISMA statement. International journal of surgery (London, England).

[66] Stroup DF, MSc P (2008). Meta-analysis of observational studies in epidemiology. JAMA.

[67] Greenland S (1987). Quantitative methods in the review of epidemiologic literature. Epidemiologic reviews.

[68] Egger M, Smith GD, Phillips AN (1997). Meta-analysis: principles and procedures. BMJ: British Medical Journal.

[69] World Cancer Research Fund/American Institute for Cancer Research. Food, Nutrition, Physical Activity, and the Prevention of Cancer: a Global Perspective. Washington DC: AICR (2007).

[70] Higgins J, Thompson SG (2002). Quantifying heterogeneity in a meta‐analysis. Statistics in medicine.

[71] Mantel N, Haenszel W (1959). Statistical aspects of the analysis of data from retrospective studies. J natl cancer inst.

[72] DerSimonian R, Laird N (1986). Meta-analysis in clinical trials. Controlled clinical trials.

[73] Egger M, Smith GD, Schneider M, Minder C (1997). Bias in meta-analysis detected by a simple, graphical test. Bmj.

[74] Begg, C. B. & Mazumdar, M. Operating characteristics of a rank correlation test for publication bias. Biometrics, 1088–1101 (1994). 7786990

